# Supplementary material for: Chiral domain walls of Mn3Sn and their memory
Source: Nat Commun. 2019 Jul 9;10:3021. doi: 10.1038/s41467-019-10815-8 (PMC6616569; doi:10.1038/s41467-019-10815-8)
Supplement: Supplementary file 1 — Supplementary Information [file 41467_2019_10815_MOESM1_ESM.pdf]

Supplemental Material for Chiral domain walls of Mn_3Sn and their memory by X. Li et al.,

Supplementary Note 1. EXPERIMENTAL METHODS

For surface magnetization measurements, we employed an array of Hall sensors based on high-mobility AlGaAs/GaAs heterostructure; The density of the two-dimensional electron gas (2DEG) was $n = 2.5 \times 10^{11}$ cm^{-2} (300 K) and it was located 160 nm below the surface. The device was fabricated using electron beam lithography and 250 V argon ions to define the mesa. Supplementary Figure 1 shows an array of ten sensors each $5 \times 5 \mu\text{m}^2$ square with a 100 μm interval between two neighboring sensors [1]. Attaching the device to the surface of the sample, the local magnetic field was determined by measuring the Hall resistivity of the sensor using an AC current source and a lock-in amplifier.

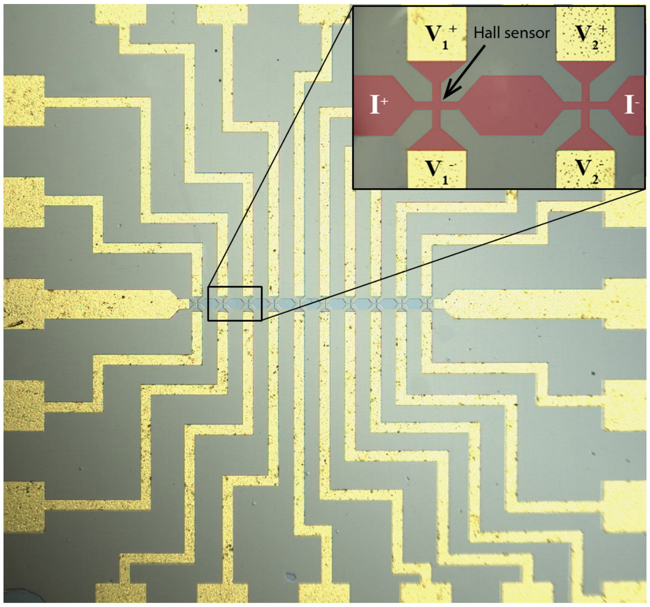

Supplementary Figure 1. Micron-size 2DEG Hall sensors: every device is consist of 10 Hall sensors, detecting the local field perpendicular to the plane.

Supplementary Note 2. SAMPLE DETAILS

For different experiments, six samples were used in this work with different aspect ratios (l_y/l_x) and cross-section shapes. The list of samples is given in Supplementary table 1. Samples #5 and #13-1 have square cross-section samples and aspect ratio (l_y/l_x) is close to unity. Samples #13-2, #13-3 and #15 have rectangular cross sections with aspect ratio (l_y/l_x) deviating from unity; The sample dubbed #triangle, has a equilateral triangle as cross section, with three sides along y axis.

	l_x(mm)	l_y(mm)	l_z(mm)	l_y/l_x
#5	0.5	0.6	2	1.2
#13-1	0.5	0.54	0.66	1.08
#13-2	0.28	0.54	0.66	1.93
#13-3	0.16	0.54	0.66	3.38
#15	1	0.2	1.8	0.2
#triangle	0.63	0.73	1.8	1.16

Supplementary table 1. Size and the aspect ratio l_y/l_x of six different samples used in this work.

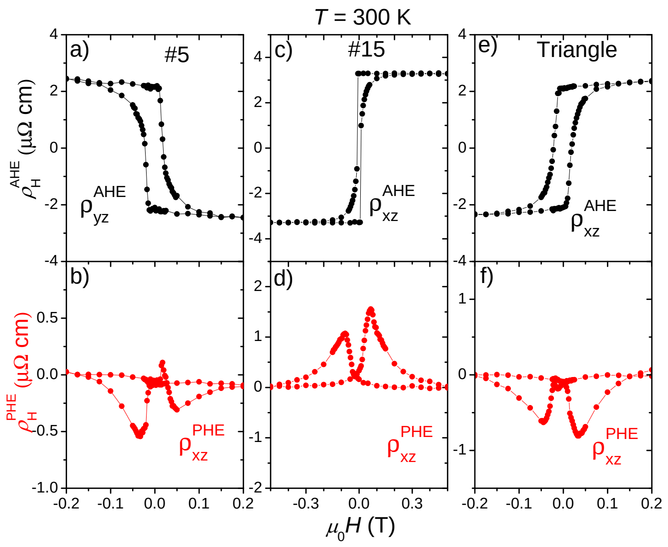

Supplementary Figure 2. Anomalous and planar Hall resistivity in the square sample #5, rectangular sample #15 and the triangle sample.

Supplementary Note 3. PLANAR HALL EFFECT (PHE) IN DIFFERENT SAMPLES

The existence of the planar Hall effect was reproduced in more than three samples and with different set-ups and cross-sections (Supplementary Figure 2). Supplementary Figure 2a and Supplementary Figure 2b represent anomalous and planar Hall resistivity in sample #5 with the magnetic field along x axis, and the electric field measured simultaneously along both x- and y-axes. Supplementary Figure 2c and Supplementary Figure 2d show anomalous and planar Hall resistivity in sample #15, the one in which the Nernst data was shown in the main text. The electric field E_x was measured for two different orientations of the magnetic field. Supplementary Figure 2e and Supplementary Figure 2f show the Hall data in a sample with triangle cross-section. The planar Hall effect is present in all these three samples and the ratio ($\rho^{\mathrm{PHE}}/\rho^{\mathrm{AHE}}$) is always between 0.3 to 0.4. It's worth noting that the width of the regime II is different and depended on the aspect ratio l_y/l_x [2].

Supplementary Note 4. EXTRACTION OF TOPOLOGICAL HALL EFFECT (THE) AND TOPOLOGICAL NERNST EFFECT (TNE)

Supplementary Figure 3a and Supplementary Figure 3d compares the hysteretic loops of the Hall and the Nernst response with the magnetization. Supplementary Figure 3b and Supplementary Figure 3e show the same comparison between normalized signals (after subtracting the high-field slope). One can see that the two responses do not scale with each other in regime II. Supplementary Figure 3c and Supplementary Figure 3f show $\rho_{xz}^{\mathrm{THE}}(\mu_0 H) = \rho_{xz}^{\mathrm{A}}(\mu_0 H) - C(M(\mu_0 H) - \mu_0 H\chi)$ and $S_{xz}^{\mathrm{TNE}}(\mu_0 H) = S_{xz}^{\mathrm{A}}(\mu_0 H) - C^*(M(\mu_0 H) - \mu_0 H\chi)$, where χ is the high-field susceptibility (the slope of the magnetization outside the hysteresis loop), $C = \rho_{xz}^{\mathrm{A}}(\mu_0 H = 0T)/M(\mu_0 H = 0T)$ and $C^* = S_{xz}^{\mathrm{A}}(\mu_0 H = 0T)/M(\mu_0 H = 0T)$ are two fitting constants.

Supplementary Note 5. TEMPERATURE DEPENDENCE OF PHE AND PNE

Supplementary Figure 4, shows the evolution of PHE and PNE as the temperature changes from 300 K to 50 K. In the whole temperature range studied, the ratios of both PHE(THE) and PNE(TNE) to AHE and ANE remain constant.

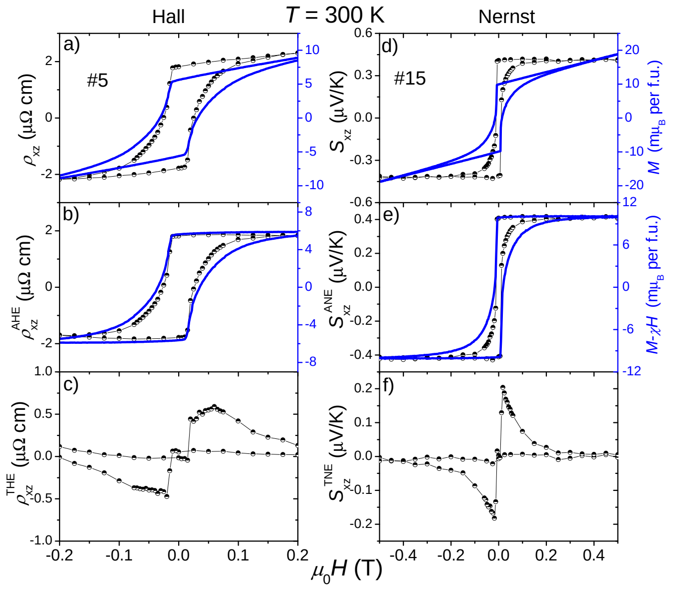

Supplementary Figure 3. (a) and (d) THe Hall and Nernst signals compared with the magnetization. (b) and (e) Comparison of the anomalous Hall and Nernst response with the magnetization subtracted the high field slope. (c) and (f) The topological Hall and Nernst response subtracted from (b) and (e).

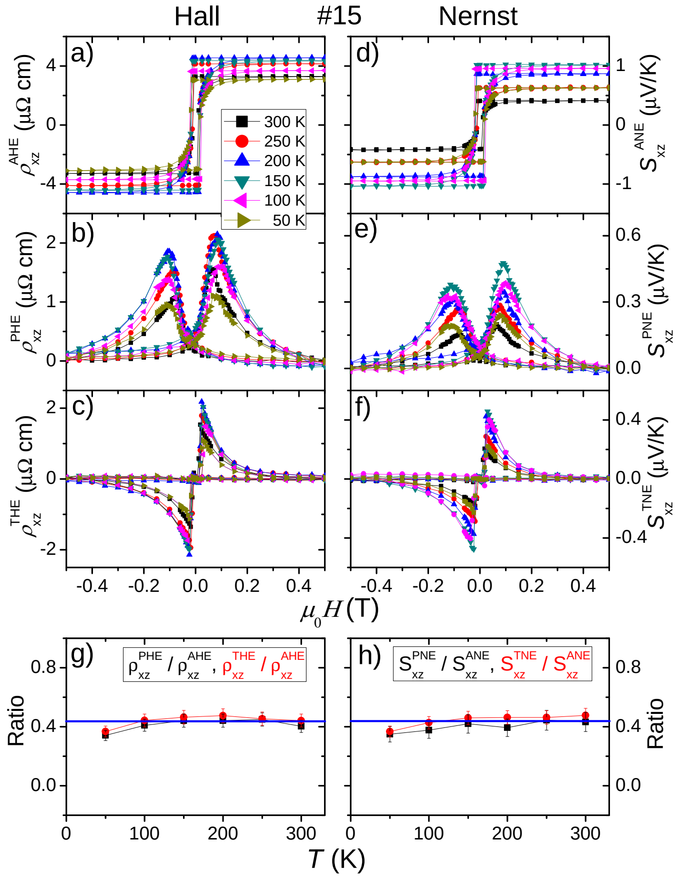

Supplementary Figure 4. Three types of Hall response (a-c) and Nernst response (d-f) with the temperature from 300K to 50K. (g) and (h) Temperature dependent ratio of $\rho_{xz}^{\rm PHE}/\rho_{xz}^{\rm AHE}$(black square left) and $\rho_{xz}^{\rm PHE}/\rho_{xz}^{\rm AHE}$ (red circle left), $S_{xz}^{\rm PNE}/S_{xz}^{\rm ANE}$(black square right) and $S_{xz}^{\rm PNE}/S_{xz}^{\rm ANE}$ (red circle right). All the ratios keep a constant near 0.4.

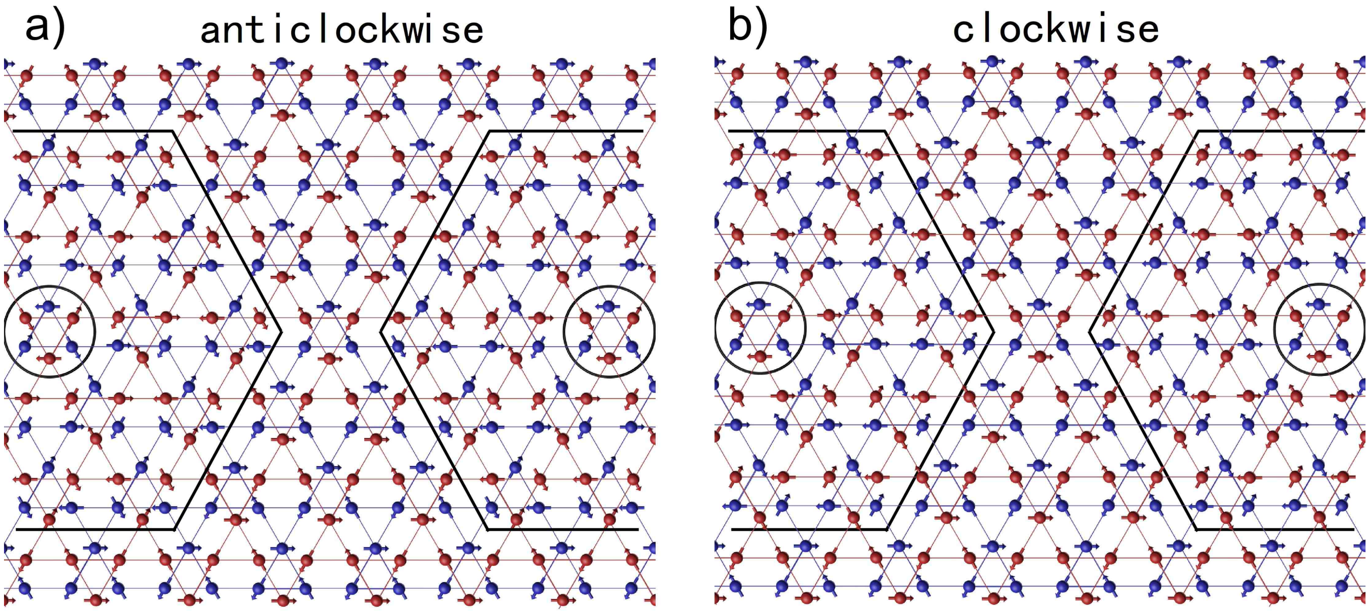

Supplementary Figure 5. Domain wall with clockwise (a) or anticlockwise (b) rotation with rectangular boundaries.

Supplementary Note 6. SPIN TEXTURE WITH RECTANGULAR BOUNDARIES

Supplementary Figure 5, shows two domains separated by a domain wall in a rectangular configuration[3].

[1] Collignon C., Superfluid density and carrier concentration across a superconducting dome: The case of strontium titanate *et al.*, Phys. Rev. B **96**, 224506 (2017).

[2] Li X., Xu L., Zuo, H., Subedi, A., Zhu, Z., & Behnia, K. Momentum-space and real-space Berry curvatures in Mn_3Sn. SciPost Phys. **5**, 063 (2018).

[3] Liu, J. & Balents, L. Anomalous Hall Effect and Topological Defects in Antiferromagnetic Weyl Semimetals: Mn_3Sn/Ge. Phys. Rev. Lett. **119**, 087202 (2017).
